# Portable Impedance Analyzer for FET-Based Biosensors with Embedded Analysis of Randles Circuits’ Spectra

**DOI:** 10.3390/s25113497

**Published:** 2025-05-31

**Authors:** Norman Pfeiffer, Martin Bach, Alice Steiner, Anna-Elisabeth Gerhardt, Joan Bausells, Abdelhamid Errachid, Albert Heuberger

**Affiliations:** 1 Fraunhofer IIS, Institute for Integrated Circuits IIS, 91058 Erlangen, Germany; martin.bach@iis.fraunhofer.de (M.B.); alice.steiner@iis.fraunhofer.de (A.S.); anna-elisabeth.gerhardt@iis.fraunhofer.de (A.-E.G.); albert.heuberger@fau.de (A.H.); 2 CSIC—Instituto de Microelectronica de Barcelona (IMB-CNM), 08193 Barcelona, Spain; joan.bausells@imb-cnm.csic.es; 3 Institut des Sciences Analytiques, Université de Lyon, 69100 Villeurbanne, France; abdelhamid.errachid-el-salhi@univ-lyon1.fr; 4 Information Technology (Communication Electronics), Friedrich-Alexander-Universität Erlangen-Nürnberg, 91058 Erlangen, Germany

**Keywords:** electrochemical impedance spectroscopy, circular fitting, FET-based biosensors, ISFET, randles circuit, impedance analyzer

## Abstract

The electrochemical impedance spectroscopy (EIS) is a measurement method for characterizing bio-recognition events of a sensor, such as field-effect transistor-based biosensors (BioFETs). Due to the lack of portable impedance spectroscopes, EIS applies mainly in laboratories preventing application-oriented use in the field. This work presents a portable impedance analyzer (PIA) providing a 4-channel EIS of BioFETs. It performs the analysis of the recorded spectra by determining the charge transfer resistance $R_{\mathrm{ct}}$ with a power-saving algorithm. Therefore, a circle is fitted into the Nyquist representation of the Randles circuit, from whose zero crossings $R_{\mathrm{ct}}$ can be determined. The introduced algorithm was evaluated on 100 simulated spectra of Randles circuits. To analyze the overall system, an adjustable reference circuit was developed that simulates configurable Randles circuits. Additional measurements with pH-sensitive ion-sensitive field-effect transistors (ISFETs) demonstrate the application of the measurement system with electrochemical sensors. Using simulated spectra, the circular fitting is able to detect $R_{\mathrm{ct}}$ with a median accuracy of 1.2% at an average nominal power of 40 mW and 3054 µs computing time. The PIA with the embedded implementation of the circuit fitting achieves a median error for R_ct_ of 4.2% using the introduced Randles circuit simulator (RCS). Measurements with ISFETs show deviations of 6.5 ± 2.8% compared to the complex non-linear least squares (CNLS), but is significantly faster and more efficient. The presented system allows a portable, power-saving performance of EIS. Future optimizations for a specific applications can improve the presented system and enable novel low-power and automated measurements of biosensors outside the laboratory.

## 1. Introduction

Miniaturized biosensors are enabling the implementation of biochemical analyses outside of traditional laboratory settings, which were previously only feasible within the confines of a laboratory environment. The application areas are extensive, whether in the health sector [1], environmental monitoring [2], food monitoring [3] or agriculture [4].

One specific type of electrochemical biosensors are impedimetric sensors, which are measured by electrochemical impedance spectroscopy (EIS). Impedimetric biosensors allow differentiated acquisition of changes in capacitance and resistance that occur on the sensor surface. Exemplary applications of impedimetric biosensors are immunosensors, deoxyribonucleic acid (DNA) sensors and enzyme-based sensors. On the one hand, Faradaic impedance spectroscopy is used to analyze electrode interfaces with respect to electron transfer processes resulting from biorecognition events. On the other hand, non-Faradaic impedance spectroscopy measures capacitance changes without redox couples [5]. Faradaic-like impedance spectroscopy is used in the application of field-effect transistor-based biosensors (BioFETs). The signal is amplified by a semiconductor, which enables highly sensitive readouts. When EIS is applied to BioFETs, its transimpedance is measured. In this case, an input voltage $\Delta v_{i}$ is applied to the counter electrode (CE), which results in a drain-source current $\Delta i_{\mathrm{DS}}$ [6]. Here, the use of EIS has been shown to provide a higher sensitivity in measuring the response of the sensor to changes in analyte concentration [7,8]. This is due to the significant changes in the impedance spectrum that can be recorded for even small variations in the threshold voltage as a result of the exponential behavior of the transconductance as a function of the gate-source voltage $g_{m}\left(V_{\mathrm{GS}}\right)$ in the subthreshold (weak inversion) operation of BioFETs [9].

Although there are many research activities and scientific publications focusing on the development of novel biosensors, it is notable that only a small proportion of biosensors have been transferred from research to market-ready products, such as Point of care (POC). One of the possible reasons for this discrepancy is that many research results cannot withstand the typical criteria necessary for ideal operations outside the laboratory and directly at an application. These criteria are known as REASSURED (real-time connectivity, ease of specimen collection, affordable, sensitive, specific, user-friendly, rapid and robust, equipment-free, and deliverable to end users) [10]. In order to meet these criteria, it is necessary not only to further develop biological receptors or transducers but also to pay attention to overall measurement systems, which, among others factors, includes the instrumentation of the biosensors. Only instrumentation that allows automated measurement and signal analysis avoids the need for trained laboratory personnel in the application scenario, which is elementary for many of the listed criteria. Thus, instrumentation is an important component through which biosensors can become ubiquitous in the future and analyses can be carried out quickly and easily. Various portable impedance spectroscopes have already been published, which achieved good accuracies despite their portable characteristics, but are not suitable for transimpedance measurements with BioFETs due to their properties. The reasons for this include the lack of a potentiostat and a driver for drain-source voltages [11,12,13,14,15,16], and a limited frequency [12] or impedance range [17]. Furthermore, all the systems mentioned cannot analyze the impedance spectra directly on the embedded system, but require appropriate personal computer (PC) software. Therefore, these systems cannot be used as standalone devices, which in turn limits the degree of automation and the simplicity of operation. Even commercially available PIAs such as CSX-64 (Sciospec Scientific Instruments GmbH, Bennewitz, Germany) or EmStat4S (PalmSens BV, Houten, Netherlands) cannot cover these properties and are therefore not suitable as stand-alone devices for BioFETs by default.

In order to enable the analysis of impedance spectra on embedded systems, Quadratic Interpolation Non-Iterative Parameter Estimation (QINIPE) is a method optimized for low-power applications [18]. It uses the imaginary part of the measured spectrum to identify a characteristic frequency at which the parameters of the equivalent circuit can be determined using closed-form expressions. However, this method is based on a simple 2R-1C model and elements such as the constant phase element (CPE) or Warburg impedance are not taken into account. Consequently, the application disqualifies various biosensors that follow the Randles circuit. Another published measurement system that allows on-board impedance analysis can only detect impedance levels using thresholds [19]. In [20], the direct determination of $R_{\mathrm{ct}}$ on a portable device is presented to diagnose SARS-CoV-2 infections. However, this work utilizes a Raspberry Pi 4B, which, as a single-board computer, offers considerably higher processing power than a microcontroller and uses its own main memory. As a result, the Raspberry Pi 4B consumes significantly more power, making it unsuitable for low-power applications. The current state of research reveals a gap in miniaturized instrumentation that allows for the measurement of impedimetric biosensors in non-laboratory settings and enables the automatic analysis of the recorded impedances directly on the embedded system.

A further challenge in the development of such instrumentation, incorporating integrated signal analysis, is the determination of a methodology for the assessment of the entire signal processing chain, both in terms of its hardware and software components. Portable impedance spectroscopes are mainly evaluated by measuring resistances or individual R-RC networks [12,13,14,17,19]. It is evident that these equivalent circuits do not accurately reflect the behavior of real impedimentric biosensors. Consequently, the investigation of novel algorithms, such as those dealing with Warburg impedances, is only possible to a limited extent.

This paper presents a portable impedance analyzer (PIA) that can measure an array of four BioFETs between 10 Hz and 200 kHz. Furthermore, it directly analyzes the acquired spectra on the embedded system using an efficient algorithm based on circular fitting. Consequently, the PIA serves as an automatic measurement system that enables the potential use of BioFETs to extend beyond the confines of a laboratory setting. The system is characterized by a test data set with simulated data and by a physical Randles circuit simulator (RCS). The RCS enables the simulation of not only 2R-1C circuits but also more complex equivalent circuit diagrams that include Warburg impedance.

An analysis of the PIA was performed based on the errors in the measurement of the impedance and the determination of charge transfer resistance $R_{\mathrm{ct}}$ as well as the computational effort. As an exemplary application, measurements were performed with ion-sensitive field-effect transistors (ISFETs) for pH measurements. Finally, the properties of the PIA presented here were compared with other impedance analyzers from published sources.

## 2. Materials and Methods

The following chapter describes both the PIA with embedded fitting algorithm as well as the approaches for evaluations based on simulated data, generated digitally and physically through an adjustable RCS, and the implementation of measurements with ISFETs.

### 2.1. Portable Impedance Analyzer

The PIA is designed specifically to carry out transimpedance measurements using EIS for BioFETs. The basic approach and the interconnection of the sensors have already been described in other publications [9,21].

**Figure 1:**
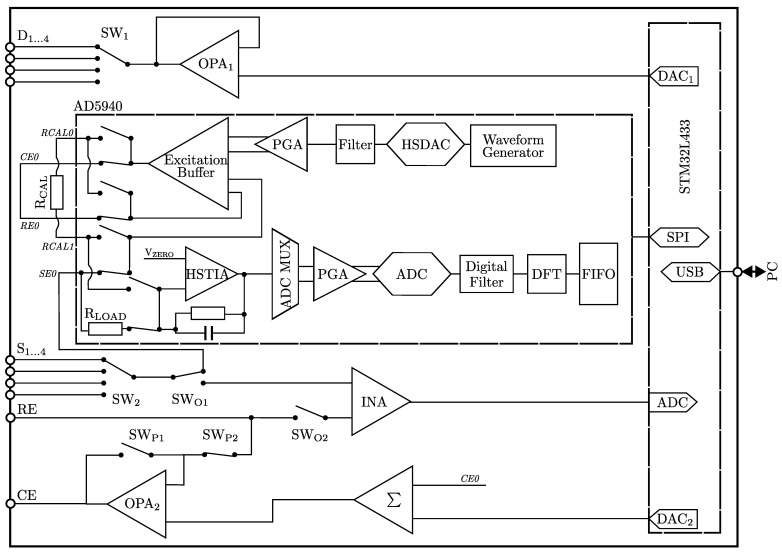
Block diagram of the portable impedance analyzer. Both internal functions of the AD5940 and external components are shown. CE0, RE0, SE0, RCAL0 and RCAL1 refer to the pin designations according to the AD5940 data sheet.

The main component of the PIA is the AD5940 (Analog Devices Inc., Norwood, MA, USA), an integrated circuit for performing various electrochemical measurement methods such as EIS. The AD5940 includes two analog front end (AFE) loops with excitation modules, potentiostats, transimpedance amplifiers (TIAs), a programmable gain amplifier (PGA) and a 16-bit analog-to-digital converter (ADC) designed for different frequency ranges with corresponding power consumption. A low-bandwidth AFE loop covers a range from direct current (DC) to 200 Hz, whereas a high-bandwidth AFE loop covers higher frequencies up to 200 kHz. For the sake of clarity, Figure 1 shows only the high-frequency loop, which includes a high speed transimpedance amplifier (HSTIA) and a high speed digital-to-analog converter (HSDAC). The sensor to be measured is connected to the internal AFE loops via a programmable switching matrix. Both the gain and the load resistance of both transimpedance amplifiers are programmable. External amplification and calibration resistors ($R_{\mathrm{CAL}}$) can also be connected to the chip through the matrix. Using external calibration resistors $R_{\mathrm{CAL}}$, the AD5940 performs a ratiometric measurement. The digitized signals are evaluated by means of the discrete fourier transform (DFT). The real and imaginary parts of the impedance can then be read out with a first-in-first-out (FIFO) buffer.

To enable multi-channel measurements of BioFETs and cover larger operating ranges for future sensor developments, an external circuit was developed for the AD5940. This is controlled by the microcontroller and enables three functions: (1) adjustment of the excitation signal with a larger offset, (2) generation of the drain-source voltage $V_{\mathrm{DS}}$ to adjust the operating point of the BioFET using ${\mathrm{DAC}}_{1}$ and a voltage follower (operational amplifier ${\mathrm{OPA}}_{1}$, AD8606), and (3) measurement of the open-circuit voltage (OCV).

To realize (1), it was necessary to add an external potentiostat (${\mathrm{OPA}}_{2}$, AD8606) to the AD5940. This potentiostat can be configured to measure two-electrode set-ups (CE and working electrode (WE)) as well as three-electrode setups (CE, reference electrode (RE) and WE). By means of a summing amplifier (Σ, AD8616), the alternating current (AC) signal of the AD5940 is superimposed with a DC voltage generated by the digital-to-analog converter ${\mathrm{DAC}}_{2}$ of the microcontroller. The DC voltage always refers to $V_{\mathrm{ZERO}}$, as this represents the potential at the source of the BioFET through the HSTIA.

The acquisition of OCV (3) was used to correct the generated gate-source DC voltage of the ISFETs and thus ensure precise adjustment of the operating point. The OCV is measured using an instrumentation amplifier (INA), AD8221 between the RE and source with a gain G=30 which is digitized by the 12-bit ADC of the microcontroller.

The PIA is powered by a universal serial bus (USB) connection. USB 2.0 also ensures communication with a PC, allowing measurement parameters to be set and measurement results to be forwarded.

The measurement of the four sensors is performed sequentially. To achieve this, the individual sensor connections are wired in an alternating sequence using analog switches (${\mathrm{SW}}_{X}$, DG9232).

The measurement process is triggered by a USB command. This includes the initialization function of the AD5940, the calibration of the AD5940 based on $R_{\mathrm{CAL}}$ and, subsequently, the actual measurement of the impedance spectrum. Here, settling processes of the electrochemical sensors are omitted due to delays between the activation of the waveform generator and digitization of measured values.

**Figure 2:**
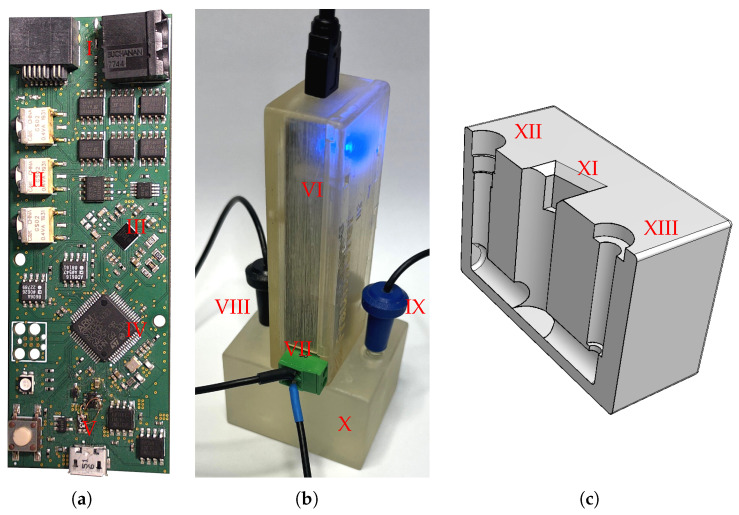
(**a**) PCB of the PIA, (**b**) the measurement setup for the application with ISFETs without opaque box and (**c**) the 3D cross-sectional model of measuring chamber. I: Connectors for ISFETs, RE and CE; II: Switches for changing between two-electrode (CE, WE) or three-electrode setup (CE, RE, WE); III: AD5940; IV: microcontroller (STM32L4): V: USB port for power and communication with a PC; VI: PIA with casing; VII: Connection for the external RE and CE VIII: CE; IX: RE; X: Measuring chamber filled with pH solution; XI: Opening for the ISFET chip; XII: Opening for RE; XIII: Opening for CE.

For the miniaturized realization of the circuit, a printed circuit board (PCB) was designed, as shown in Figure 2a. The size of the PIA is 90 × 33 mm. it can be recognized that CPE leads to a rotation of the circle around the coordinate origin. The exponent n determines the angle α = n$\frac{\pi }{2}$ between the tangent of the semicircle, which passes through the origin and the x-axis [22]. Considering this characteristic, a circular fitting can be used instead of an elliptical fitting for the analysis of the Randles circuit, which is presumably more resource-efficient with fewer degrees of freedom.

### 2.2. Fitting Algorithm

Once the spectrum has been measured, the data are usually analyzed using elaborate fittings to determine the model parameters of the Randles circuit. For this purpose, commercial laboratory software such as ZView (Scribner LLC, Southern Pines, NC, USA) or EC-Lab (BioLogic, Seyssinet-Pariset, France) is typically used, which are generally based on complex non-linear least squares (CNLS) fitting [23]. Recent articles [24,25] presented a simplified elliptical fitting method as an alternative to CNLS for evaluating EIS spectra.

**Figure 3:**
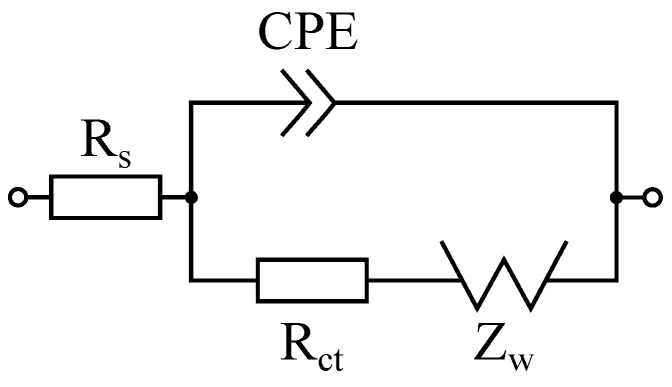
The fitting algorithm assumes the shown equivalent circuit diagram (ECD) of the biosensor after a Randles circuit.

When a Randles circuit is given for a specific biosensor (see Figure 3), a semicircle appears in the Nyquist plot, created by the CPE and $R_{\mathrm{ct}}$. This semicircle intersects the x-axis of the Nyquist plot (real part, Re(Z)) at two points: at high frequencies, the intersection corresponds to the solution resistance $R_{s}$, while the other intersection indicates $R_{s}$ + $R_{\mathrm{ct}}$. However, this second intersection is typically not straightforward to read, as the spectrum in this low frequency range is predominantly influenced by the Warburg impedance $Z_{W}$. Characterized by a constant phase of 45°, $Z_{W}$ represents the influence of diffusion to or from an electrode. A theoretical electrode with an infinitely large surface area, allowing for unrestricted diffusion, can be described using a prefactor $Q_{w}$:

(1) $$\begin{array}{cc} Z_{w}\left(f\right)=\frac{1}{Q_{w}\sqrt{\left(i2\pi f\right)}} & . \end{array}$$

When an elliptical and a circular fitting are geometrically applied to the spectrum in the Nyquist diagram, the larger intersection of the ellipse and circle, respectively, with the x-axis represents the resistance value for $R_{s}+R_{\mathrm{ct}}$. $R_{\mathrm{ct}}$ is the most relevant parameter for biosensors based on faradaic EIS [26], which can also be applied for BioFETs [9]. In [23], the elliptical fitting was also used to address changes in the semicircle due to a CPE. It should be noted, however, that a CPE does not compress the semicircle. Rather, through the formula

(2) $$\begin{array}{cccc} Z_{CPE}\left(f\right) & =\frac{1}{Q{\left(i2\pi f\right)}^{n}} & \; & n\in [0,1] \end{array}$$

The following description of the circular fitting algorithm was implemented on a STM32L4 using the C programming language. The general idea is to first describe the spectrum using a polynomial fitting and thus identify the relevant part of the spectrum for the circular fitting. Then the circular fitting can be applied to the spectrum, whereby $R_{s}$ and $R_{\mathrm{ct}}$ could be geometrically determined by the intersection points of the circle with the x-axis.

**Figure 4:**
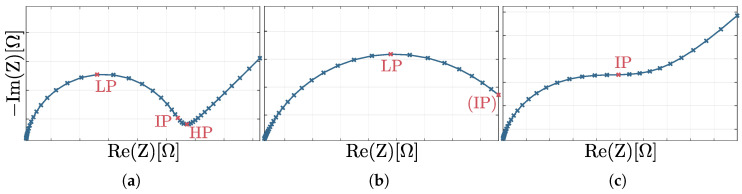
Classification of the spectra into three categories ((**a**) category I; (**b**) category II; (**c**) category III) based on the occurrence of high point (HP), low point (LP) and inflection point (IP).

In the first step, a fifth-degree polynomial was fitted according to the principle of least squares, assuming that the imaginary part was a function of the real part. This procedure has the advantage that the graphs to be examined are smoothed and can be subjected to a curve discussion in the Nyquist plot. At this point, the curve discussion allows the identification of the relevant range of the spectrum for the circular fitting. The aim is to utilize the high-frequency sections of the semicircle, as they are minimally impacted by the Warburg impedance. Therefore, the low points (LPs) are determined considering the change of sign of the first derivative. This allows the spectrum to be categorized as shown in Section 2.3 and Figure 4. For categories I and II, the LP is used as the cutoff point and all higher frequency measurement points are used for the circular fitting. If no LP is found, a category III spectrum is given and the inflection point (IP) is identified by the second derivative. For the delimitation of the area relevant for the circular fitting, the turning point with the lowest real part, i.e., the highest frequency, is selected. For better performance, the first and second derivatives of the polynomial were precalculated as far as possible and hard coded. If no IP can be found either, all data points are used for the circular fitting.

The circular fitting was realized with the Newton-Taubin method [27,28] giving the coordinates of the centre point of the circle $\left(x_{0},y_{0}\right)$ and the radius r as output. Subsequently, the zero crossings can be determined using the general circle equation

(3) $${\left(x-x_{0}\right)}^{2}+{\left(y-y_{0}\right)}^{2}=r^{2}$$

with y=0. This results in the equation of the resistance $R_{s}$:

(4) $$R_{s}=\frac{2x_{0}-\sqrt{{\left(2x_{0}\right)}^{2}-4\left(x_{0}^{2}+y_{0}^{2}-r^{2}\right)}}{2}.$$

$R_{\mathrm{ct}}$ is obtained by subtracting $R_{s}$ from the right intersection of the circle:

(5) $$R_{ct}=\frac{2x_{0}+\sqrt{{\left(2x_{0}\right)}^{2}-4\left(x_{0}^{2}+y_{0}^{2}-r^{2}\right)}}{2}-R_{s}.$$

In addition to implementing the circuit fitting on the PIA, which enables application to real measurements (see results in Section 3.2 and Section 3.4), the algorithm was also executed on the NUCLEO-L433RC-P development board (STMicroelectronics N.V., Schiphol, The Netherlands). On the one hand, the NUCLEO development board has the advantage that the algorithm can be investigated independently of the PIA by sending simulated data from Section 2.3 via universal asynchronous receiver/transmitter (UART) to the microcontroller, and, on the other hand, it allows the simple connection of a shunt resistor for measuring the power consumption (see Section 3.3).

### 2.3. Test Data Set

100 spectra of Randles circuits were simulated and subsequently used to evaluate the circular fitting. The parameters and conditions that were used have already been described in previous work [24]. The advantage of the simulated test data is that the underlying values of the parameters are known as nominal values, and therefore the error caused by the fitting algorithms can be determined directly.

For the development of the circular fitting, the simulated data was divided into three categories based on the occurrence of HPs, LPs and IPs. Thus, these categories describe different manifestations of the Randles circuit in the Nyquist diagram, as shown in Figure 4. Category I shows a HP, LP and IP, respectively. In contrast, category II has only a LP and optionally a IP. Category III exclusively includes an IP.

### 2.4. Randles Circruit Simulator

**Figure 5:**
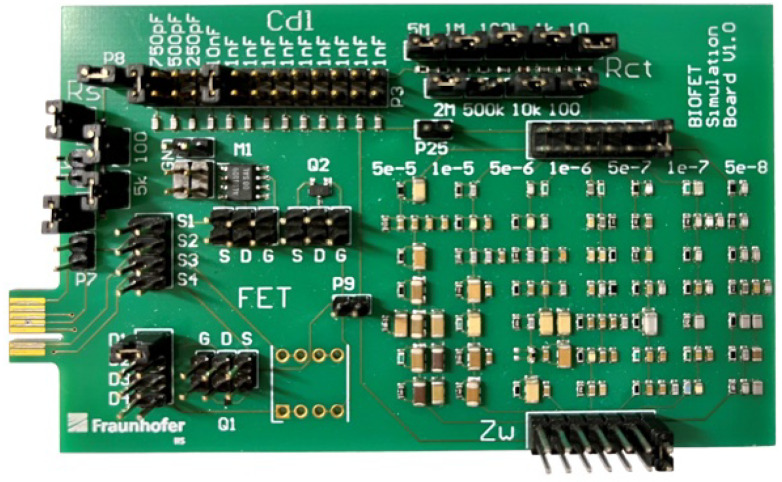
PCB of the RCS. All parameters ($R_{\mathrm{ct}}$, $R_{s}$, $C_{\mathrm{dl}}$, $Z_{w}$) can be set via jumpers. The connector is compatible with the presented PIA, and thus its performance can be examined by comparing target and calculated values.

To evaluate the portable impedance spectroscope and the circular fitting implemented on the microcontroller, an equivalent circuit of the Randles circuit was realized on a PCB, as shown in Figure 5. For this purpose, this reference system replaced the BioFET. Although the complete Randles circuit, like any other ECD for non-oscillatory electrochemical processes, can be modeled by a serial concatenation of parallel RC elements [29], this is not suitable for developing a physical simulator for validation purposes. It is due to the circuit complexity and the fact that the target values for individual parameters cannot be set directly. Although the electrical behavior can be simulated by cascading parallel RC elements, the parameter values of the Randles circuit cannot be set independently of each other, and therefore do not allow the target values to be determined directly. The advantage of this simulator is that the impedance spectroscope including the implemented algorithms can be tested with known nominal values of the parameters, and thus an error determination for the appropriate equivalent circuit can be performed.

**Figure 6:**
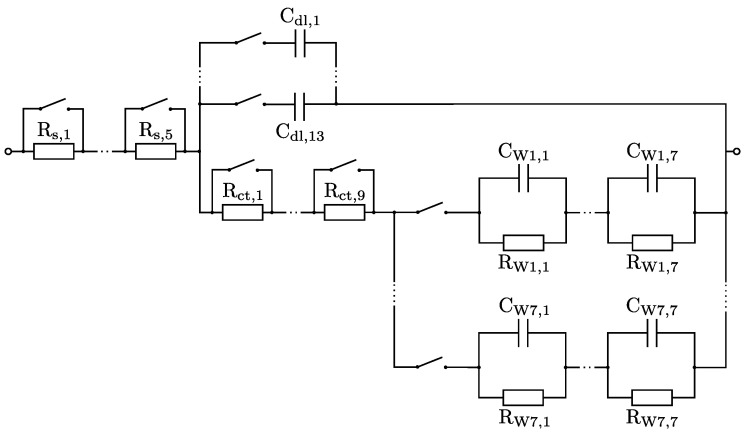
Circuit diagram of the RCS. $R_{s}$, $R_{\mathrm{ct}}$ and $C_{\mathrm{dl}}$ are simulated by series or parallel connection of several components. Seven different Warburg impedance elements are used individually and are simulated by RC networks.

The circuit of the RCS is shown in Figure 6. The PCB allowed the solution resistance $R_{s}$ and charge transfer resistance $R_{\mathrm{ct}}$ to be simulated by serial resistors with jumpers. The double-layer capacitance $C_{\mathrm{dl}}$ can also be adjusted using parallel capacitors using jumpers. The Warburg impedance $Z_{W}$ describes diffusion effects and cannot be reproduced by single passive electrical components:

(6) $$\begin{array}{cc} Z_{w}\left(f\right)=\frac{1}{2Q_{w}\sqrt{\left(i\pi f\right)}} & . \end{array}$$

**Figure 7:**
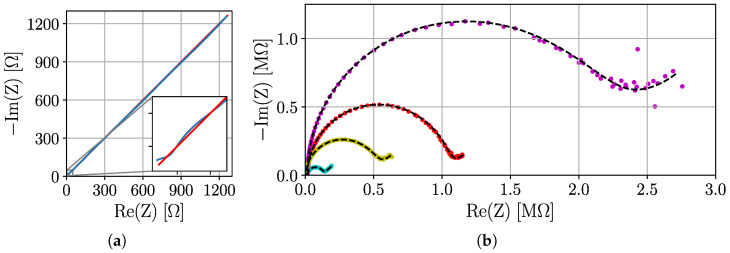
Nyquist plots of RCS measurements with the BioLogic VSP-300 potentiostat. (**a**) Comparison of a theoretical curve (red) of a Warburg impedance ($Q_{w}=5\times 10^{-5}$) with the measured curve (blue) of the simulated Warburg impedance. Relative errors mainly occur in the high frequency respectively low impedance range. (**b**) Exemplary Randles circuits comparing the theoretic (black) and the measured simulation curves. The following configurations ($R_{s}$, $C_{\mathrm{dl}}$, $Q_{w}$, $R_{\mathrm{ct}}$) are used: 6.11 kΩ, 750pF, 500 nS·$\sqrt{s}$, 500 kΩ (yellow), 16.11 kΩ, 250 pF, 500 nS·$\sqrt{s}$, 1.01 MΩ (red), 16.11 kΩ, 750 pF, 1 μS·$\sqrt{s}$, 111.11 kΩ (cyan), 15.00 kΩ, 250 pF, 100 nS·$\sqrt{s}$, 2.11 MΩ (magenta).

However, it is possible to approximate $Z_{W}$ by a serial concatenation of parallel RC elements [29]. By using more RC pairs, the approximation of the Warburg impedance is more accurate. To simulate a semi-infinite $Z_{W}$, 7 parallel connected RC elements were used in series. The number of RC elements is a compromise between circuit complexity and accuracy and is based on previous LT-Spice simulations described in Appendix A. For the Warburg impedance as well as for the entire circuit, resistors of the E96 series (1% tolerance) and capacitors of the E24 series (5% tolerance) were chosen. Since these E series do not cover every intermediate value, the optimal values determined for simulating the Warburg impedance had to be rounded when selecting the components. Simulations have shown that this leads to an error of ±0.17% in fitting the Warburg impedance. In the Supplementary Material, the determined root mean squared relative error (RMSRE) between the theoretical spectra of the Warburg impedance elements and the equivalent circuits with components of the E series is presented, both in LT-Spice simulations and in real measured spectra with the BioLogic VSP-300 potentiostat. Exemplary results of the latter are also shown in Figure 7b. The measured spectra were fitted using CNLS [23] to trace $Q_{w}$ of the Warburg impedance elements. $Q_{w}=5\times 10^{-5}$ was not used for evaluations of the PIA because of the large error of −8.0%, which is mainly caused by the high-frequency range (see Figure 7a. The maximum error is −4.3% for all other values of $Q_{w}$.

### 2.5. Real-World Tests with ISFETs

In order to evaluate the PIA with the embedded circular fitting under practical conditions, measurements were carried out with pH-sensitive ISFETs. The measurement set-up is shown in Figure 2b. The fundamentals and operation of the ISFETs used for real-world tests have been published elsewhere [9], as well as the corresponding fabrication process [30]. ISFETs with an active gate area of 20 µm × 400 µm are utilized, featuring a gate dielectric of 100 nm of silicon nitride on top of 78 nm of silicon dioxide. The silicon chip contains four ISFETs and is mounted on a simple PCB designed for immersion in liquid. Wire bonding connects the chips and the bonding wires, along with the edges of the chip, are coated with an epoxy-based resist (EPO TEK H70; Epoxy Technology, Billerica, MA, USA).

A measurement chamber was fabricated using additive manufacturing techniques (see Figure 2c. The components were produced with a Formlabs Form 3 3D printer, utilizing RS-F2-GPCL-04 Clear Resin photopolymer selected for its transparency and durability. The chamber’s design includes three connected compartments, arranged to accommodate the RE, CE, and the ISFETs. The total liquid volume capacity is approximately 4 mL.

Samples with pH values of 5.09, 7.07, and 8.67 were analyzed. The solutions were prepared by initially measuring the pH of a mixture comprising 5 mM of tris-(hydroxymethyl) methylamine and 0.4 M of magnesium nitrate $\mathrm{Mg}{\left({\mathrm{NO}}_{3}\right)}_{2}$ using a pH meter. Thereafter, the initial solution was divided into three smaller aliquots, the pH of which was adjusted using hydrochloric acid (HCl) and then validated by a control measurement.

Before the impedance measurement, the ISFETs were placed in the solution for 10 min to achieve electrochemical equilibrium. This was also performed as protected from light irradiation by a box to avoid photoelectric effects. All measurements were performed in an air-conditioned room at 20±2°C. EIS was performed between 10 Hz and 100 kHz using an AC sinusoidal signal with an amplitude of $V_{\mathrm{GS},\mathrm{AC}}=75\;\mathrm{mV}$. The drain-source voltage was set to $V_{\mathrm{DS}}=500\;\mathrm{mV}$ and the gate-source offset voltage to $V_{\mathrm{GS},\mathrm{DC}}=0\;\mathrm{mV}$.

## 3. Results

The following chapter presents the results of the investigations described in Section 2.

### 3.1. Evaluation of the PIA

**Figure 8:**
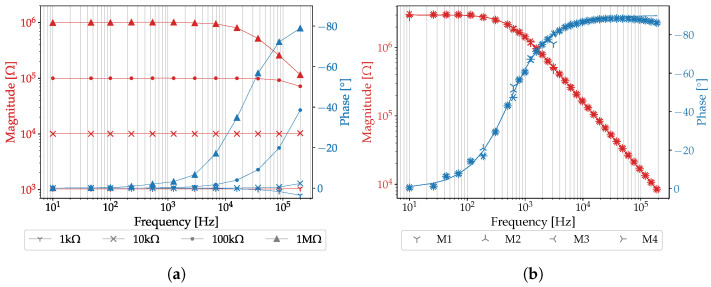
Frequency response (red: magnitude; blue: phase) of the portable impedance analyzer. (**a**) Frequency responses considering resistors to be measured between 1 kΩ and 1 MΩ. (**b**) Frequency responses of a 3 MΩ resistor in parallel connection with a 100pF capacitor with four repetitions (M1–M4). The solid line shows the theoretical frequency response.

Figure 8a shows the Bode diagrams measured through single resistors. The spectra clearly show that low-pass behavior occurs depending on the resistor value. The deviations in the high-frequency range when measuring the resistances are caused by the input capacitances of the transimpedance amplifier and by parasitic capacitances of measuring leads. Although the switches were selected in such a way that there is minimal influence on the signals (3.8 pF switch-off and 7.8 pF switch-on-capacitances), they are not included in the calibration, which in turn could also lead to deviations, especially in the high-frequency range. However, this behavior is less relevant for measuring biosensors that follow the Randles equivalent circuit, as high impedance is not expected at high frequencies. In the low-frequency range <100Hz, accuracies of 2.6% at 1 kΩ, 0.6% at 10 kΩ, 0.3% at 100 kΩ and 0.7% at 1 MΩ were obtained.

With four sequential measurements of a 3 MΩ resistor in parallel with a 100pF capacitor, Figure 8b shows that the PIA provides good repeatability of the measurements. The average repeatability error to the median value of the individual measurement frequencies is −0.2±1.2%.

### 3.2. Accuracy and Robustness of Circular Fitting

In order to investigate the fitting algorithm on the microcontroller independently of the PIA, the accuracies for the determination of $R_{\mathrm{ct}}$ were first analyzed using simulated data (see Section 2.3). Here, the development board NUCLEO-L433RC-P was used. The median error achieved of $R_{\mathrm{ct}}$ was 1.2±2.8%. A total of 95 out of 100 simulated spectra could be fitted, while the rest could not be fitted successfully.

**Figure 9:**
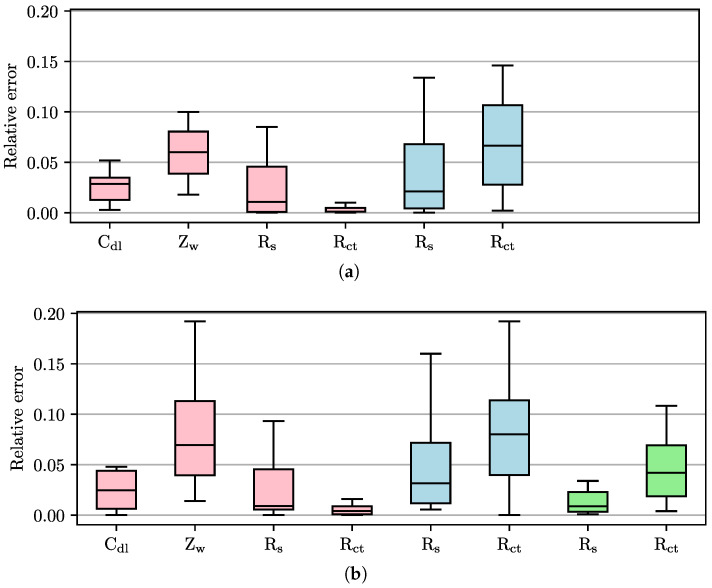
Relative error of the determined parameters of the Randles circuit using different instrumentations and different fitting methods. The instrumentation was (**a**) BioLogic VSP-300 and (**b**) PIA. Fitting methods used were CNLS (red), elliptical fitting (blue) and circular fitting (green). Since circular fitting was only implemented on the microcontroller, it could not be investigated with the VSP-300. 24 random configurations of the RCS were measured. Outliers are not shown for better illustration.

To investigate the circular fitting, the RCS from Section 2.4 was measured with the PIA as well as the BioLogic VSP-300 potentiostat. The RCS configurations are chosen randomly. The data was analyzed using CNLS [23], elliptical fitting [24,25] and circular fitting presented in this work. The results are shown in Figure 9 as relative errors of the determined parameter values to the target values. $Z_{w}$ and $C_{\mathrm{dl}}$ cannot be determined by ellipses or circular fittings, respectively. Since the circular fitting was specifically implemented as embedded programming, it was not examined in combination with the VSP-300.

Figure 9 clearly shows that the results of the PIA (errors for $R_{\mathrm{ct}}$: CNLS 0.4%, elliptical fitting 8.0%) are marginally worse than those of the VSP-300 (CNLS 0.1%, elliptical fitting 6.7%). For $Z_{W}$, the medians are of similar order of magnitude, but the interquartile range of the relative errors is larger using the PIA. With a median error of 4.2% for $R_{\mathrm{ct}}$, the circular fitting shows clearly reduced errors compared to the elliptical fitting. This phenomenon can be attributed to the fact that the appearance of the Randles circuit in the Nyquist plot is described by a (half) circle. Furthermore, the CPE leads to a rotation of the circle with the zero point as the fixed point, rather than an apparent compression of the circle. However, the semicircle can be distorted on the low-frequency side due to the influence of the Warburg impedance, which can lead to errors due to the larger number of degrees of freedom of ellipses. Nevertheless, the circular fitting does not achieve the same level of accuracy as the CNLS.

### 3.3. Computing Effort of the Circular Fitting on the Embedded System

**Figure 10:**
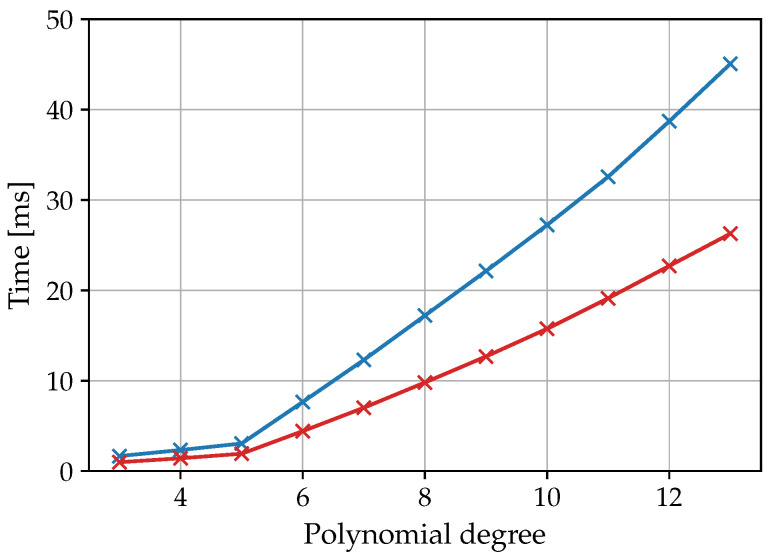
Influence of the polynomial degree and the number of spectral data points (blue: 50 data points, red: 25 data points) on the calculation time of the entire fitting algorithm. The recorded times are the mean values from 100 spectra of the test data set.

Both computing time and power consumption were determined from the execution of the polynomial fitting to the final calculation of $R_{\mathrm{ct}}$. The time measurement was recorded via the system tick time (SysTick) of the microcontroller, through which a measurement accuracy of 12.5ns could be achieved at a clock rate of $f_{\mathrm{clock}}=80\;\mathrm{MHz}$. Therefore, spectra from the test data set with 50 and 25 data points and different degrees of polynomial fitting (see Figure 10) were investigated. It is evident that the required computing time became significantly for polynomial degrees above the fifth degree. This in turn reinforces the choice of the fifth-degree polynomial, also with regard to efficiency. The total duration of the overall fitting algorithm with preceding fifth-degree polynomial fitting lasted, on average 3054.28 µs for spectra with 50 data points. The total time is composed of the polynomial fitting (76%), the calculation of the derivatives and the determination of the extreme and inflection points (21%) and the circular fitting (3%).

The implementation presented here is therefore significantly more efficient than the previously published elliptical fitting [25], which requires 2 ms calculation time with only 20 data points on a PC (1.90 GHz Intel^®^ Core^TM^ i7-8650U processor with 16 GB DDR4-3200-SDRAM) using the Python 3.10 programming language. In this research, the CNLS method took 460 ms.

The current consumption of the microcontroller during fitting was measured through a shunt resistor $R_{\mathrm{shunt}}=12$ Ω. The power consumption was tested with firmware that does not use communication modules (e.g., UART, direct memory access (DMA)). Instead, data from exemplary spectra were hard coded in the firmware and applied during the measurement of the current consumption. During the calculation of the fitting, an average current consumption of 12mA at 3.3V could be estimated, which corresponds to a power consumption of approximately 40mW. Taking into account the calculation time of the algorithm, this results in an energy consumption of 40mW·3.01ms=120.4µJ. However, meaningful comparison of the energy consumption of the algorithm with that of the entire PIA is challenging, as the PIA is supplied via USB and communicates permanently with the PC. This results in the microcontroller utilizing almost no low-power modes due to interrupts, thereby leading to a high overall consumption. The PIA should be regarded as an intermediate version and can be adapted for specific applications by leveraging wireless communication technologies. For instance, Bluetooth Low Energy (BLE) requires only approximately 0.1 J for 20 communication cycles [31] and enables the microcontroller to enter a sleep state for most of the time. In the current setup, the PIA requires 26.87 J for a measurement with 30 logarithmically distributed measurement points and a measurement duration of 56.8 s. Of this, 2.57 J are accounted for by all components of the AFE (AD5940, OPAs, switches). Consequently, the energy consumption of the algorithm is considerably lower than the power consumption of the actual measurement.

### 3.4. Real-World Tests with ISFETs

**Figure 11:**
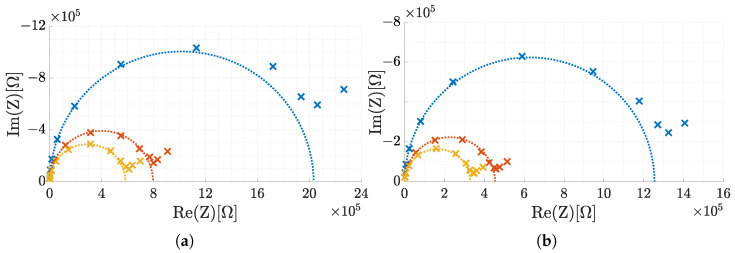
Nyquist plot of two ISFETs (**a**,**b**) for pH5.09 (yellow), pH7.07 (orange) and pH8.67 (blue). The result of the circular fitting was plotted in addition to the measurement points.

Figure 11 shows the Nyquist plots of two ISFETs placed simultaneously in three different pH solutions. The data were analyzed with the circular fitting, CNLS with PreFit [23] and the elliptical fitting [24,25]. Assuming that CNLS is the reference method in this case, the circular fitting achieved an average error of 6.5±2.8% in the determination of $R_{\mathrm{ct}}$. In comparison, the elliptical fitting achieved an error of 7.0±2.5%.

## 4. Discussion and Conclusions

This paper presented a PIA that can perform four-channel EIS for BioFETs and analyze Randles circuits spectra directly onboard via a microcontroller by means of circular fitting. Using simulated data and the introduced RCS, the accuracies of the PIA could be determined with regard to the impedance measurement and for the determination of $R_{\mathrm{ct}}$. To the best of the authors’ knowledge, no such system currently exists, either in academic research or as a commercial product. A comparison of the PIA presented in this paper with other published works is provided in Table 1. Here, other PIA for EIS that perform single sine analysis were considered.

The AFE of the PIA offers a cost-effective solution, with a price of approximately 42 €. This is significantly more economical than employing two potentiostats, such as the AD5940, to produce gate-source and drain-source voltages for four channels, which would ultimately amount to about 88 €.

The RCS enabled investigations with biosensor-like circuits and thus made the quantitative investigation of the entire system possible in the first place. However, in future versions it could imitate more realistic sensor behaviour by modelling CPE instead of $C_{p}$ [39] and also other Warburg impedance elements besides the semi-infinite type [40]. This would allow a more comprehensive evaluation of novel algorithms for the analysis of spectra characterized by Randles circuits.

The PIA presented is capable of measuring EIS for biosensors that follow the Randles circuit. As expected, parasitic capacitances made it impossible to measure high impedances in higher frequency ranges. However, this characteristic should have little impact when using biosensors that follow the Randles equivalent circuit, as the impedance tends to minimize with increasing frequency. When using the PIA with very high-impedance biosensors, it is important to consider this characteristic, as the circular fitting particularly includes the spectrum in the high-frequency range.

The circular fitting presented enables the analysis of impedance data directly on the embedded device. It achieves better accuracies than the elliptical fitting and is even more efficient. Although the circular fitting cannot achieve the accuracies of the CNLS, it has significant advantages, e.g., for stand-alone measurement systems without a PC or applications where energy efficiency plays an important role.

The accuracy of the determination of $R_{\mathrm{ct}}$ by the PIA was determined using different methods. The fitting of simulated spectra (I) on the microcontroller resulted in an error of 1.2%, whereas the use of the RCS (II) achieved an error of 4.2% and the application on real ISFETs (III) resulted in an error of 6.5%. This multi-stage approach investigates different properties of the PIA, which is why the varying results are comprehensible. In (I), the accuracy of the embedded algorithm is examined independently of the hardware components of the PIA, as the target values of the simulated spectra are known and these are only superimposed by Gaussian noise. Measurement technology components do not yet play a role here. In (II), the accuracy of the entire PIA is examined, which includes not only the algorithm but also the measurement technology for performing the EIS. Thus, the measurement result is influenced by measurement inaccuracies, such as systematic measurement errors, parasitic capacitances, or frequency-dependent errors. The investigation with ISFETs (III) proves the applicability of the PIA to real sensors, and is influenced by other sources of error such as sensor drifts or non-ideal spectral behavior. With (III), it should be noted that, in contrast to (I) and (II), the target value is not given but must be determined by applying CNLS. This in turn can also lead to inaccuracies in the method. For the interpretation of the results, it should be emphasized that the ranges of individual ECD parameters were established based on the transimpedances of the present ISFETs [9] and their application as BioFETs [8,21,41,42]. Considering future applications of the PIA in combination with other biosensors, it is suggested to perform studies with adapted parameter ranges.

The accuracy of the parameter determination by the circular fitting is similar to QINIPE [18], but offers the advantage of working with the more complex Randles circuit instead of a simple 2R-1C model. Compared to QINIPE, which requires a calculation time of 9.2 ms for 29 frequencies, the circular fitting is also the faster method for analyses. In principle, the circular fitting can also be transferred to other ECDs, e.g., to those that have two or more time constants and thus several semicircles in the Nyquist diagram, as for example in [43]. The applicability of this method is limited in cases where the semicircle of $R_{\mathrm{ct}}$ and CPE cannot be clearly delimited from the other semicircle, for example by a HP analogous to Figure 4a. In such cases, the separation of the relevant frequency range will be unsuccessful, or the circular fitting will be influenced by the second semicircle. Furthermore, the same principle applies if the Warburg impedance dominated the semicircle to such an extent that there was no IP in the Nyquist plot. This assertion also applies to the different types of Warburg impedances [40], which is why their impact on the behavior of the circular fitting should be further examined.

The experiments were conducted in a controlled laboratory environment at a constant room temperature. Future research should incorporate varying temperature conditions to explore potential dependencies across different application scenarios. Additionally, it is essential to investigate the long-term stability of the PIA in combination with specific BioFETs within these application contexts.

The presented device facilitates the automated measurement and analysis of spectra, rendering it especially advantageous for biosensor applications operating on low power. To illustrate, a portable biosensor can continuously monitor an analyte and transmit data via wireless or cellular communication only when meaningful changes in the analyte concentration are detected. This capability is crucial because communication typically demands more power and can substantially limit data throughput, for instance, when utilizing satellite communications.

## Figures and Tables

**Table 1:** Comparison of various impedance analyzers with this work. The frequency range (f range), impedance range (Z range), number of channels (${\#}_{\mathrm{ch}}$), ability to measure BioFETs, accuracy of magnitude and phase measurements, test circuits used for validation and the manner in which the model parameters were determined are considered. It should be noted that the test conditions for determining the accuracy vary between the different works. “n.i.” stands for “no information”.

Source	f Range [Hz]	Z Range [Ω]	${\#}_{\mathrm{ch}}$	BioFET-Ready	Accuracy (mag., ph.)	Test Circuit	Parameter Determination
[32]	10 m–100 k	100–10 G	1	no	5%, 3°	$R_{1}+R_{2}\left|\right|C_{1}$	no
[33]	10–100 k	n.i.	1	no	1.9%, 2.4%	$R_{1}+R_{2}\left|\right|C_{1}$	PC (ZView)
[34]	10 m–50 k	n.i.	1	no	5%, 7°	$R_{1}+R_{2}\left|\right|C_{1}$	no
[35]	2–1.4 k	200–1 M	1	no	n.i.	$R_{1}\left|\right|C_{1}$	no
[20]	0.5–10 k	n.i.	1	no	n.i.	$R_{1}+R_{2}\left|\right|C_{1}$	Raspberry Pi, model fit
[36]	1–100 k	20–1 M	1	no	3.5%, 2.8°	$R_{1}+C_{1}+R_{2}\left|\right|C_{2}\left|\right|L_{1}$	PC (ZView)
[37]	10 m–100 k	100–10 G	1	no	5%, 3°	/	n.i.
[38]	1 m–100 k	1–1 G	1	no	1%, 0.1°	$R_{1}+R_{2}\left|\right|C_{1}$	no
[13]	1–100 k	100–1 M	1	no	1%, 1.5°	$R_{1}+R_{2}\left|\right|C_{1}$; $R_{1}||(R_{2}+R_{3}\left|\right|C_{1})$	no
[11]	10 m–100 k	10–10 G	1	no	2%, 2.5°	$C_{1}||(R_{1}+R_{2}\left|\right|C_{2})$	no
[12]	1 k–100 k	9–18 M	1	no	2%, n.i.	L; R	no
[14]	10–100 k	n.i.	1	no	1.1%, 1.3°	$R_{1}+R_{2}\left|\right|C_{1}$	no
[15]	100–50 k	10–100 k	1	no	2.5%, 1°	($R_{1}\left|\right|C_{1})+(R_{2}||(C_{2}+R_{3}$)) + $\left(R_{4}||C_{3}\right)$	no
[16]	10–2 M	100–200 k	8	no	1.2%, 1.8°	R	no
[17]	5–100 k	10–100 k	1	no	n.i.	$R_{1}\left|\right|C_{1}$	PC
This work	10–200 k	1 k–1 M	4	yes	2.6%, 1°	$R_{1}$; $R_{1}\left|\right|C_{1}$; $R_{1}+(C_{1}\left|\right|\left(R_{2}+Z_{W}\right))$	STM32L4, circular fit

**Figure A1:**
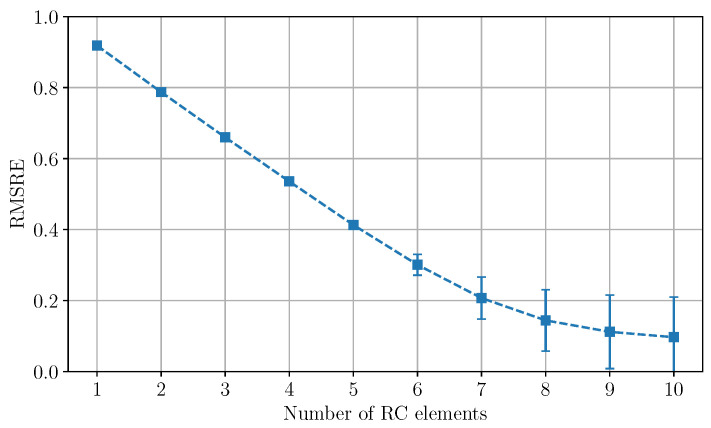
RMSRE of 200 spectra of $Z_{W}$ fitted by RC circuits with different numbers of elements using CNLS.

## Data Availability

The raw data supporting the conclusions of this article will be made available by the authors on request.

## Supplementary Materials

The following supporting information can be downloaded at: https://www.mdpi.com/article/10.3390/s25113497/s1.

## Abbreviations

The following abbreviations are used in this manuscript:

AC	Alternating current
ADC	Analog-to-digital converter
AFE	Analog front end
BioFET	Field-effect transistor-based biosensor
BLE	Bluetooth low energy
CE	Counter electrode
CNLS	Complex non-linear least squares
CPE	Constant phase element
DAC	Digital-to-analog converter
DC	Direct current
DFT	Discrete fourier transform
DMA	Direct memory access
DNA	Deoxyribonucleic acid
ECD	Equivalent circuit diagram
EIS	Electrochemical impedance spectroscopy
FET	Field effect transistor
FIFO	First-in-first-out
GUI	Graphical user interface
HP	High point
HSDAC	High speed digital-to-analog converter
HSTIA	High speed transimpedance amplifier
INA	Instrumentation amplifier
IoT	Internet of things
IP	Inflection point
IQR	Interquartil range
ISFET	Ion-sensitive field-effect transistor
LP	Low point
OCV	Open-circuit voltage
OPA	Operational amplifier
PC	Personal computer
PCB	Printed circuit board
PGA	Programmable gain amplifier
PIA	Portable impedance analyzer
POC	Point of care
QINIPE	Quadratic interpolation non-iterative parameter estimation
RCS	Randles circuit simulator
RE	Reference electrode
RMSRE	Root mean squared relative error
SysTick	System tick time
TIA	Transimpedance amplifier
UART	Universal asynchronous receiver/transmitter
USB	Universal serial bus
WE	Working electrode

## Appendix A. Number of RC Elements for the Simulation of Z_W_

To determine an appropriate number of RC elements to imitate $Z_{W}$ for the RCS, the spectra of 200 random $Z_{W}$ were generated between 10 Hz and 200 kHz. From these spectra, CNLS [23] was used to determine the associated parameter values. Instead of the Warburg impedance, 1–10 serially connected RC elements were used as equivalent circuits iteratively. The calculation of the RMSRE was used to determine which number of RC elements is appropriate. Finally, seven RC elements were chosen for the RCS, as this led to an acceptable circuit complexity and an average RMSRE of 0.21. At this point, the decline in RMSRE slows down significantly with increasing complexity (Figure A1).

## References

[1] Tu J., Torrente-Rodríguez R.M., Wang M., Gao W. (2020). The Era of Digital Health: A Review of Portable and Wearable Affinity Biosensors. Adv. Funct. Mater..

[2] Gavrilas S., Ursachi C.S., Perța-Crisan S., Munteanu F.D. (2022). Recent Trends in Biosensors for Environmental Quality Monitoring. Sensors.

[3] Ferrari A.G.M., Crapnell R.D., Banks C.E. (2021). Electroanalytical Overview: Electrochemical Sensing Platforms for Food and Drink Safety. Biosensors.

[4] Kundu M., Krishnan P., Kotnala R., Sumana G. (2019). Recent developments in biosensors to combat agricultural challenges and their future prospects. Trends Food Sci. Technol..

[5] Katz E., Willner I. (2003). Probing Biomolecular Interactions at Conductive and Semiconductive Surfaces by Impedance Spectroscopy: Routes to Impedimetric Immunosensors, DNA-Sensors, and Enzyme Biosensors. Electroanalysis.

[6] Kharitonov A.B., Wasserman J., Katz E., Willner I. (2001). The Use of Impedance Spectroscopy for the Characterization of Protein-Modified ISFET Devices: Application of the Method for the Analysis of Biorecognition Processes. J. Phys. Chem. B.

[7] Zayats M., Huang Y., Gill R., Ma C.a., Willner I. (2006). Label-Free and Reagentless Aptamer-Based Sensors for Small Molecules. J. Am. Chem. Soc..

[8] Ben Halima H., Bellagambi F.G., Hangouët M., Alcacer A., Pfeiffer N., Heuberger A., Zine N., Bausells J., Elaissari A., Errachid A. (2023). A novel electrochemical strategy for NT-proBNP detection using IMFET for monitoring heart failure by saliva analysis. Talanta.

[9] Bausells J., Ben Halima H., Bellagambi F.G., Alcacer A., Pfeiffer N., Hangouët M., Zine N., Errachid A. (2022). On the impedance spectroscopy of field-effect biosensors. Electrochem. Sci. Adv..

[10] Land K., Boeras D., Chen X.S., Ramsay A., Peeling R. (2018). REASSURED diagnostics to inform disease control strategies, strengthen health systems and improve patient outcomes. Nat. Microbiol..

[11] Hoja J., Lentka G. Portable analyzer for impedance spectroscopy. Proceedings of the XIX IMEKO World Congress Fundamental and Applied Metrology.

[12] Breniuc L., David V., Haba C.G. Wearable impedance analyzer based on AD5933. Proceedings of the 2014 International Conference and Exposition on Electrical and Power Engineering (EPE).

[13] Buscaglia L., Carmo J., Oliveira O. (2023). Simple-Z: A low-cost portable impedance analyzer. IEEE Sens. J..

[14] Ibba P., Crepaldi M., Cantarella G., Zini G., Barcellona A., Petrelli M., Abera B.D., Shkodra B., Petti L., Lugli P. FruitMeter: An AD5933-Based Portable Impedance Analyzer for Fruit Quality Characterization. Proceedings of the 2020 IEEE International Symposium on Circuits and Systems (ISCAS).

[15] Jiang Z., Yao J., Wang L., Wu H., Huang J., Zhao T., Takei M. (2019). Development of a Portable Electrochemical Impedance Spectroscopy System for Bio-Detection. IEEE Sens. J..

[16] Ye X., Jiang T., Ma Y., To D., Wang S., Chen J. (2023). A portable, low-cost and high-throughput electrochemical impedance spectroscopy device for point-of-care biomarker detection. Biosens. Bioelectron. X.

[17] Al-Ali A., Elwakil A., Ahmad A., Maundy B. Design of a portable low-cost impedance analyzer. Proceedings of the International Conference on Biomedical Electronics and Devices.

[18] Simic M., Babic Z., Risojević V., Stojanovic G. (2020). Non-Iterative Parameter Estimation of the 2R-1C Model Suitable for Low-Cost Embedded Hardware. Front. Inf. Technol. Electron. Eng..

[19] Sawhney M.A., Conlan R. (2019). POISED-5, a portable on-board electrochemical impedance spectroscopy biomarker analysis device. Biomed. Microdevices.

[20] Perdomo S.A., Ortega V., Jaramillo-Botero A., Mancilla N., Mosquera-DeLaCruz J.H., Valencia D.P., Quimbaya M., Contreras J.D., Velez G.E., Loaiza O.A. (2021). SenSARS: A Low-Cost Portable Electrochemical System for Ultra-Sensitive, Near Real-Time, Diagnostics of SARS-CoV-2 Infections. IEEE Trans. Instrum. Meas..

[21] Ben Halima H., Bellagambi F.G., Brunon F., Alcacer A., Pfeiffer N., Heuberger A., Hangouët M., Zine N., Bausells J., Errachid A. (2023). Immuno field-effect transistor (ImmunoFET) for detection of salivary cortisol using potentiometric and impedance spectroscopy for monitoring heart failure. Talanta.

[22] Besançon G., Becq G., Voda A. (2020). Fractional-Order Modeling and Identification for a Phantom EEG System. IEEE Trans. Control Syst. Technol..

[23] Pfeiffer N., Jechow M., Wachter T., Hofmann C., Errachid A., Heuberger A. (2021). Impact of normalization, standardization and pre-fit on the success rate of fitting in electrochemical impedance spectroscopy. Curr. Dir. Biomed. Eng..

[24] Pfeiffer N., Wachter T., Frickel J., Hofmann C., Errachid A., Heuberger A. Elliptical Fitting as an Alternative Approach to Complex Nonlinear Least Squares Regression for Modeling Electrochemical Impedance Spectroscopy. Proceedings of the 14th International Joint Conference on Biomedical Engineering Systems and Technologies.

[25] Pfeiffer N., Wachter T., Frickel J., Halima H.B., Hofmann C., Errachid A., Heuberger A., Gehin C., Wacogne B., Douplik A., Lorenz R., Bracken B., Pesquita C., Fred A., Gamboa H. (2021). Determination of Charge Transfer Resistance from Randles Circuit Spectra Using Elliptical Fitting. Proceedings of the Biomedical Engineering Systems and Technologies.

[26] Kemp N.T. (2021). A Tutorial on Electrochemical Impedance Spectroscopy and Nanogap Electrodes for Biosensing Applications. IEEE Sens. J..

[27] Taubin G. (1991). Estimation of Planar Curves, Surfaces, and Nonplanar Space Curves Defined by Implicit Equations with Applications to Edge and Range Image Segmentation. Pattern Anal. Mach. Intell. IEEE Trans..

[28] Chernov N.I., Lesort C. (2005). Least Squares Fitting of Circles. J. Math. Imaging Vis..

[29] Schönleber M., Ivers-Tiffée E. (2015). Approximability of impedance spectra by RC elements and implications for impedance analysis. Electrochem. Commun..

[30] Vozgirdaite D., Ben Halima H., Bellagambi F.G., Alcacer A., Palacio F., Jaffrezic-Renault N., Zine N., Bausells J., Elaissari A., Errachid A. (2021). Development of an ImmunoFET for Analysis of Tumour Necrosis Factor-a in Artificial Saliva: Application for Heart Failure Monitoring. Chemosensors.

[31] Liu Y., Riba J.R., Moreno-Eguilaz M. (2023). Energy Balance of Wireless Sensor Nodes Based on Bluetooth Low Energy and Thermoelectric Energy Harvesting. Sensors.

[32] Grassini S., Corbellini S., Angelini E., Ferraris F., Parvis M. (2015). Low-Cost Impedance Spectroscopy System Based on a Logarithmic Amplifier. IEEE Trans. Instrum. Meas..

[33] Wang X., Zhao H., Wang A., Dong Z., Fan Y., Zhai Z. (2021). A Portable Impedance Spectroscopy Measurement Method Through Adaptive Reference Resistance. IEEE Access.

[34] Sebar L.E., Iannucci L., Angelini E., Grassini S., Parvis M. (2021). Electrochemical Impedance Spectroscopy System Based on a Teensy Board. IEEE Trans. Instrum. Meas..

[35] Tabrizi H.O., Salahandish R., Jalali P., Khalghollah M., Haghayegh F., Sanati-Nezhad A., Ghafar-Zadeh E. (2023). A Low-Cost Handheld Reconfigurable Impedimetric Readout System for Diagnostics of Viral Infections. IEEE Trans. Instrum. Meas..

[36] Chabowski K., Piasecki T., Dzierka A., Nitsch K. (2015). Simple Wide Frequency Range Impedance Meter Based on AD5933 Integrated Circuit. Metrol. Meas. Syst..

[37] Grassini S., Corbellini S., Parvis M., Angelini E., Zucchi F. (2018). A simple Arduino-based EIS system for in situ corrosion monitoring of metallic works of art. Measurement.

[38] Piasecki T., Chabowski K., Nitsch K. (2016). Design, calibration and tests of versatile low frequency impedance analyser based on ARM microcontroller. Measurement.

[39] Valsa J., Vlach J. (2013). RC models of a constant phase element. Int. J. Circuit Theory Appl..

[40] Barbero G., Lelidis I. (2017). Analysis of Warburg’s impedance and its equivalent electric circuits. Phys. Chem. Chem. Phys..

[41] Ben Halima H., Bellagambi F.G., Alcacer A., Pfeiffer N., Heuberger A., Hangouët M., Zine N., Bausells J., Elaissari A., Errachid A. (2021). A silicon nitride ISFET based immunosensor for tumor necrosis factor-alpha detection in saliva. A promising tool for heart failure monitoring. Anal. Chim. Acta.

[42] Ben Halima H., Zine N., Nemeir I.A., Pfeiffer N., Heuberger A., Bausells J., Elaissari A., Jaffrezic-Renault N., Errachid A. (2024). An ImmunoFET Coupled with an Immunomagnetic Preconcentration Technique for the Sensitive EIS Detection of HF Biomarkers. Micromachines.

[43] Ohno R., Ohnuki H., Wang H., Yokoyama T., Endo H., Tsuya D., Izumi M. (2013). Electrochemical impedance spectroscopy biosensor with interdigitated electrode for detection of human immunoglobulin A. Biosens. Bioelectron..

